# Cellular and immune response in fatal COVID-19 pneumonia

**DOI:** 10.11604/pamj.2024.49.130.45739

**Published:** 2024-12-19

**Authors:** Sylvia Nikolaeva Genova, Mina Miroslavova Pencheva, Tsvetana Ivanova Abadjieva, Nikolay Georgiev Atanasov

**Affiliations:** 1 Department of General and Clinical Pathology, Medical Faculty, Medical University Plovdiv, Plovdiv, Bulgaria,; 2 St George University Hospital, Plovdiv, Bulgaria,; 3 Department of Medical Physics and Biophysics, Faculty of Pharmacy, Medical University of Plovdiv, Plovdiv, Bulgaria,; 4 Department of Dermatology and Venereology, Medical University of Plovdiv, Plovdiv, Bulgaria,; 5 Department of Health Management and Health Economics, Faculty of Public Health, Medical University Plovdiv, Plovdiv, Bulgaria

**Keywords:** COVID-19, pneumonia, CD4+, CD8+, IgG4

## Abstract

**Introduction:**

the severity of COVID-19, causing fatal pneumonia, acute respiratory distress syndrome (ARDS), and thrombotic complications, is linked to intense inflammation. Elevated CD4+ and CD8+ cells in the lungs indicate harmful inflammation in severe cases. This study investigates immune responses in lung tissues of deceased patients across different stages of COVID-19 pneumonia.

**Methods:**

lung tissues from 160 fatal COVID-19 cases, diagnosed via Real-Time RT-PCR, were histologically analyzed to identify pneumonia stages. Inflammatory cell counts were assessed immunohistochemically. Non-parametric tests analyzed categorical variables, while regression analysis evaluated relationships between continuous variables.

**Results:**

the average patient age was 68.1 years (± 12.6). Microscopic analysis identified four pneumonia stages. CD4+, CD68 (macrophages), and IgG4 levels peaked by day 14, with notable elevation within seven days of symptom onset. CD4+ levels were significantly lower in DAD pneumonia (49.4% ± 15.7%) compared to ARDS (66.4% ± 19.3%) and thrombosis (70.2% ± 28.9%) (p < 0.05). Male patients had higher CD4+ values (68.5% ± 21.1%) than females (56.9% ± 22.4%) (p < 0.05). B cells (CD20) and NK cells were depleted across all stages. IgG4 expression reached 80-90% in acute phases but was nearly absent during organization and fibrosis stages.

**Conclusion:**

a sharp decline in CD4+ and CD8+ during acute pneumonia and sepsis reflects immune exhaustion, while their elevation in ARDS and thrombosis likely triggers cytokine storms, causing severe lung damage. Elevated IgG4 levels in acute lung tissue correlate with fatal outcomes in severe COVID-19.

## Introduction

The severity of COVID-19, leading to fatal pneumonia, ARDS and other organ damage, is associated with an intense and prolonged protective inflammatory response known as cytokine storm syndrome. The described severe cases of COVID-19 tend to have low levels of T helper and T suppressor lymphocytes, as well as memory T cells [1]. The last experience from SARS-CoV-1 and MERS suggests that T cells are the primary regulatory mechanisms of disease [2], but in SARS-CoV-1 infection, elevated levels of antibodies and cells were found to be associated with significant inflammation, cytokine storm and worsened clinical outcomes [3].

Cytotoxic and helper T lymphocytes as well as NK cells play a major role in regulating an effective antiviral response against severe acute respiratory syndrome coronavirus 2 (SARS-CoV-2) [4]. The role of CD8+ cytotoxic T cells is to recognize viral peptides presented by MHC-class-I molecules on infected cells and initiate cytotoxic damage. However, patients with SARS-CoV-2 infection often exhibit a marked decrease in the total number of NK and CD8+ T cells [5].

In patients with COVID-19, the lungs are thought to show an abundance of CD4+ and CD8+ T cells compared to normal tissue. This indicates inflammation that can be devastating in severe cases. The large number of CD68-labeled macrophages in the infected lungs contributes to respiratory distress and the characteristic “ground-glass opacity” appearance seen radiographically in the lungs. An increased amount of these immune cells is thought to lead to increased cytokine production, perpetuating the cycle of severe inflammation.

Relatively little is known about the role of T cells in Long COVID-19 (LC). More research articles in this area are shedding light on the laboratory and clinical manifestations of prolonged inflammation, secondary infections, and compromised immunity in LC [6]. The aim of this study is to investigate the immune and cellular responses on tissue sections from the lungs of deceased patients at different stages of COVID-19 pneumonia.

## Methods

**Study design and settings:** this retrospective study examined 160 deceased patients who suffered severe complications of COVID-19 resulting in fatality. Autopsies were conducted at the St George University Hospital and MHAT “St. Panteleimon” Plovdiv, Bulgaria. Autopsy material collection spanned from February 28, 2020, to May 2022, covering cases from the first, second, third and fourth waves of the pandemic, with predominant involvement of the alpha, beta, delta and omicron variants of the SARS-CoV-2 virus. For this period in Plovdiv, 113 656 patients fell ill, and 2996 (5.7%) patients passed away. Autopsied cases were 386. Of these patients, we included in the study patients with a clear pulmonary picture, a precisely established period of the onset of the disease and a positive PCR test. Exclusion criteria - overlapping stages of pneumonia, negative test for COVID-19, autopsies outside the announced options. For the statistical analysis, we divided the deceased patients into groups according to causes of death, complications, concomitant diseases, gender and age (I group<59 yo, II group>60 yo). The ethics committee has approved the research for the entire project: COVID-19 on a National Project N° KP - 06-K1/33/23.06.2020 “COVID-19 HUB - Information, Innovations and Implementation of Integrative Research activities in Bulgaria” Medical University Plovdiv, Bulgaria. Ethical committee N°R1584/23.06.21, MU-Plovdiv.

**Study population:** out of the 160 patients included in the study, severe acute respiratory syndrome (SARS) developed 90 patients as a complication of COVID-19 pneumonia. Most patients experienced complications after 14 days of illness, with 106 patients requiring mechanical ventilation. Additionally, 26 patients died in the emergency department, and the complication developed within hours in those cases, before being placed on mechanical ventilation. The earliest development of the syndrome was observed on days 7-10 from the onset of the disease.

**Genetic testing:** one hundred and fifty-eight (158) of the cases were diagnosed using a Polymerase chain reaction (PCR) test, specifically the Accu Power® SARS-CoV-2 Real-Time RT-PCR Kit (Bioneer, South Korea). Two cases were confirmed via rapid antigen testing. Remarkably, none of the deceased patients had received any doses of the COVID-19 vaccines.

### Histological examination

**Autopsy procedure:** samples were extracted from both lungs, encompassing both central and peripheral regions of the lobes. Four paraffin blocks were collected from each lung for routine examination using Hematoxylin and Eosin (HE) staining. Autopsy materials were fixed in 10% neutral buffered formalin, followed by standard processing and HE stains. Immunohistochemical analysis was performed on samples obtained from these same blocks.

**Immunohistochemistry:** immunohistochemical testing was performed on formalin-fixed, paraffin-embedded 5-µm sections. The sections were incubated for a minimum of 20 min at 90°C and after-wards were deparaffinized. Immunohistochemically, both lungs were examined with CD3 (Clone-BC33) (Dako Omnis, Santa Clara, US), CD20 L26 (Abcam, Cambridge, UK) to detect T and B cell responses, CD4+ (Clone-4B12) (Dako Omnis, Santa Clara, US) for helper T lymphocytes and CD8+ (Clone-C8/144B) (Dako Omnis, Santa Clara, US) for cytotoxic T lymphocytes, CD68 KP-1 (Abcam, Cambridge, UK) for macrophages, recombinant IgG4 (EP4420) IgG4, (Abcam, Cambridge, UK) 1: 3000. Immunohistochemical (IHC) staining was performed by Autostainer Link 48 (Dako, Agilent Technologies Inc., Glostrup, Den-mark). We use optimized reagents for Autostainer Link 48 with FLEX Ready-to-Use Anti-bodies and EnVision FLEX Visualization Systems.

Images were visualized and captured with a digital camera mounted on a Nikon Eclipse 80i microscope using NIS-Elements Advanced Research Software version 4.13 (Nikon Instruments; Tokyo, Japan). The results were reported quantitatively with morphometric measurement of positive cells, reflected as a percentage. Depending on the quantity of infiltrating immune cells, we classified the infiltration as weak (≤10%), moderate (11-49%), and strong (≥50%) infiltration of inflammatory cells. The analysis was conducted using the software system of the Leica DM6 B Fully Automated Upright Microscope System for Life Science Research.

**Statistical analysis:** results are presented with the mean and standard deviation (mean ± SD). The check for normality of biomarker frequency distribution was conducted Shapiro-Wilk Test, which is appropriate for small samples. Mean values for biomarker levels in groups by categorical variables were compared with non-parametric tests like Kruskal-Wallis Test and Wilcoxon Signed Ranks Test. The relationship between quantitative variables was assessed by single-factor linear regression models. Statistical analysis was performed on IBM SPSS Statistics (v.23). The statistical significance was considered at p < 0.05. Intervention studies that involve animals or humans, and other studies that require ethical approval, must list the authority that provided approval and the corresponding ethical approval code.

## Results

Out of 160 autopsy patients, 58 (36.2%) were men. Most patients were over 60 years of age, 137 (82.2%) with ages ranging from 28 to 88 years, an average age of 68.1 years (68.1±12.613) and a median of 71 years. The frequency distribution of patients by age differs significantly from the normal distribution (Shapiro-Wilk Sig. < 0.05). This is mainly due to the presence of six extremely low values - below 31 years of age. The significant number of cases was in the group over 60 years, with 137 (83%) patients (Table 1).

**Table 1:** demographic and health characteristics of observed cases

		CD4+ and CD8+, % in lung tissue in cohort group				
		N	Mean CD4+	SD CD4+	Mean CD8+	SD CD8+
**Total**		160	64.28	22.253	61.91	24.163
Complications	ARDS	90	66.39	19.296	61.92	24.04
	DAD	12	49.42	15.693	44.00	23.187
	Fibrosis	18	63.50	31.407	62.56	22.035
	Thrombosis	22	70.23	28.945	70.05	23.651
	Sepsis	18	57.17	14.901	63.22	24.402
	K-W Sig		0.002		0.046	
Variant of Virus	Delta	120	62.98	22.145	62.06	24.627
	Omicron	40	68.18	22.401	61.48	23.011
	M-W Sig		0.144		0.816	
Atherosclerosis	yes	66	63.17	22.653	61.28	24.813
	no	47	67.39	21.882	62.33	22.944
	complicated	47	61.02	22.268	61.96	25.648
	K-W Sig		0.306		0.929	
Arterial Hypertension	no	106	63.97	22.988	62.16	23.528
	yes	54	64.89	20.929	61.43	25.583
	M-W Sig		0.824		0.749	
Liver cirrhosis	no	144	63.91	22.882	61.49	24.823
	yes	15	67.33	16.136	63.87	16.040
	M-W Sig		0.862		0.708	
CIHD	no	84	64.55	22.652	61.88	21.784
	yes	76	63.99	21.950	61.95	26.694
	M-W Sig		0.981		0.947	
Diabetes Mellitus	no	143	62.43	22.167	60.12	24.366
	yes	17	79.82	16.512	77.00	16.194
	M-W Sig		0.001		0.008	
Gender	Female	102	56.88	22.441	57.81	27.938
	Male	58	68.49	21.118	64.25	21.528
	M-W Sig		0.001		0.103	
Age	<60 age	23	73.52	9.214	68.48	15.480
	≥ 60 age	137	62.73	23.416	60.18	25.205
	M-W Sig		0.072		0,119	

Legend: ARDS - Acute Respiratory Distress Syndrome; DAD - Diffuse Alveolar Damage; CIHD - Chronic Ischemic Heart Disease; SD - Standard Deviation; K-W Sig - Kruskal-Wallis Significance; M-W Sig - Mann-Whitney Significance;

**Macroscopically:** lung changes were bilateral and severe in all examined cases. The lungs were enlarged in size and weight (650-750g), with increased density, hyperemia and hemorrhages. Upon sectioning, their surfaces appeared homogeneous, diffusely consolidated with a brown-reddish color. Nodular, dense areas were observed predominantly subpleurally.

**Figure 1:**
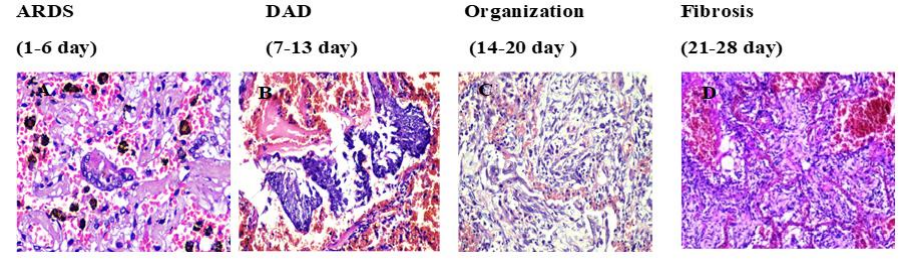
stages in the development of COVID-19 pneumonia: A) ARDS, giant multinucleated cells and diffuse damage to alveolar septa, HEx20; B) DAD, formation of syncytial structures, HEx20; C) organization with fresh granulation tissue, HEx20; D) collagen proliferation and fibrosis, HEx20

**Microscopically:** we have identified four stages in the development of COVID-19 pneumonia. In the course of the study, we found that the different stages developed within specific time frames. Detailed pulmonary changes are outlined in our other publications [7]. In Stage I (1-6 days), the lungs had pronounced hyperemia, dilated capillaries in the septa, and numerous macrophages presented in the alveoli. In this stage, ARDS developed in severe cases before the appearance of specific morphological changes. Stage II (7-13 days) was characterized by the desquamation of alveolocytes and their viral transformation into giant mononuclear cells, accompanied by the formation of syncytial structures and DAD (Diffuse Alveolar Damage), (Figure 1A, B). In stage III (14-20 days), early organization was marked by diffuse capillary proliferation with fresh granulation tissue. (Figure 1C). Stage IV (21-28 days) entails the development of fibrosis and collagen formation (Figure 1D). In the third and fourth stages, the causes of death are the complete obliteration of the alveoli, blocking of the respiratory units and a strong decrease in oxygen saturation which results in asphyxia in the patients.

**Figure 2:**
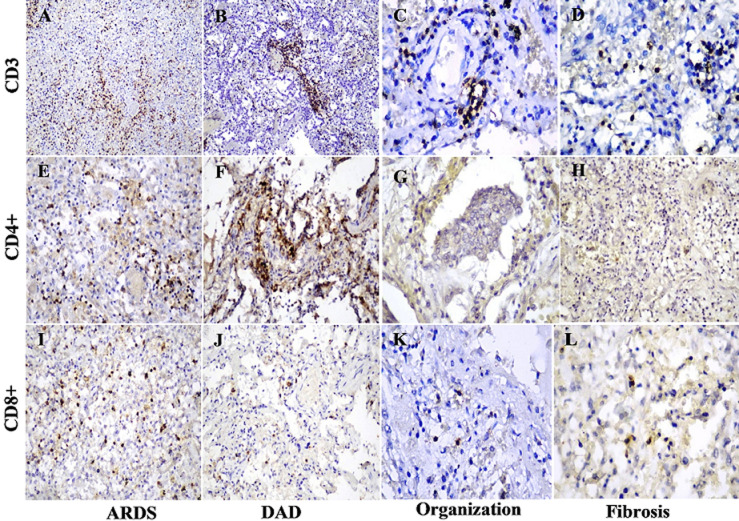
immunohistochemical staining of CD3, CD4+, CD8+ cells in lung tissue: A, B) CD3 expression in 70-80% in the acute phase of the disease, x10; C, D) CD3 drops to 20-30% in the organizing phase and fibrosis, x20; E, C) CD4+ expression in 50-60% in the acute phase, x20; G, H) CD4+ lack of expression in the organizing phase and fibrosis, x20; I, J) CD8+ with 10-20% expression in the acute phase, x20; K, L) CD8+ reduced to single cells in the phase of organization and fibrosis, x40

**Figure 3:**
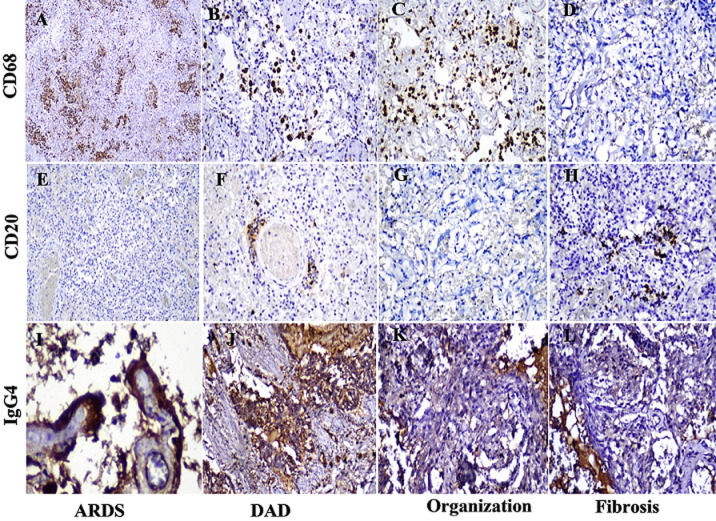
immunohistochemical staining of CD68, CD20, IgG4 cells in lung tissue: A, B, C) CD68 expression in macrophages represented up to 80% in the phases of respiratory distress, acute pneumonia and early organization, x10, x20; D) in the fibrosis phase, macrophages are depleted x20; E, G) CD20, B-lymphocytes are almost completely exhausted in the ARDS stage, x10 F) DAD: in the stage of desquamative pneumonia, we observed a scant amount of B lymphocytes around small vessels with microthrombi, x20; G) CD20 complete absent in organization phase, x10; H) CD20 weakly expressed in the fibrotic stage, x20; J, G) IgG4 intense expression up to 80-90% in lung tissue in the acute phase, x40, x20; K, L) IgG4 almost complete depletion in the phases of organization and fibrosis, x20

**Immunohistochemistry presented the infiltration by the different types of immune cells in the four consecutive stages of DAD pneumonia:** CD4+ cells are presented in (66.39% ± 19.3%) in the stage of ARDS, thrombosis (70.23% ± 28.95%) and lower in DAD pneumonia (49.42% ± 15.69%) (p < 0.05). We observed extreme increases in CD68-positive macrophages and IgG4 within day 7 of symptoms and peaked at day 14. In the stage of organization and fibrosis, the number of inflammatory cells and immunoglobulins, such as CD8+, CD4+ helper cells, CD68-positive macrophages, and IgG4 - sharply decreases. B cell lymphocytes, labeled with CD20 is almost completely exhausted during the acute stage of pneumonia and weakly expressed in the fibrotic stage. In the stage of desquamative pneumonia, we observed a scant amount of B lymphocytes around small vessels with microthrombi (Figure 2, Figure 3).

**Comparisons of CD4+ and CD8+:** the continuous variables CD4+ and CD8+ are not normally distributed (p < 0.05). However, the mean of CD4+ (64.28 ± 22.25) is higher than those of CD8+ (61.91 ± 24.16). In the case of CD4+, statistically significant dependence was established (R2 = 0.06; p < 0.05) with a regression coefficient βage = -0.431, which is for one unit change in age, CD4+ will decrease by 0.431 percentage points. The regression model of CD8+ with independent variable age is statistically insignificant and has a very low slope coefficient estimate (R2 = 0.003; p > 0.05; βage = -0.098). Means and standard deviations of CD4+ and CD8+, grouped by categorical variables are summarized in Table 1.

**Figure 4:**
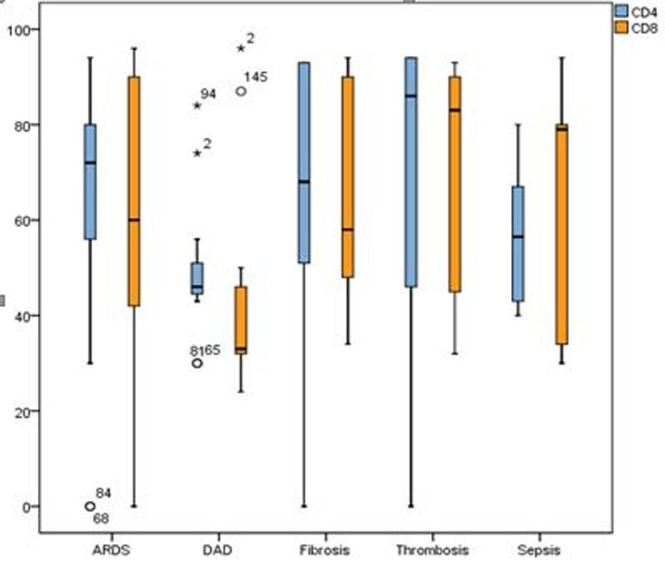
dependence on CD4+ and CD8+ by complications; high mean CD4+ and CD8+ expression levels, except for the group with sepsis and DAD

**Figure 5:**
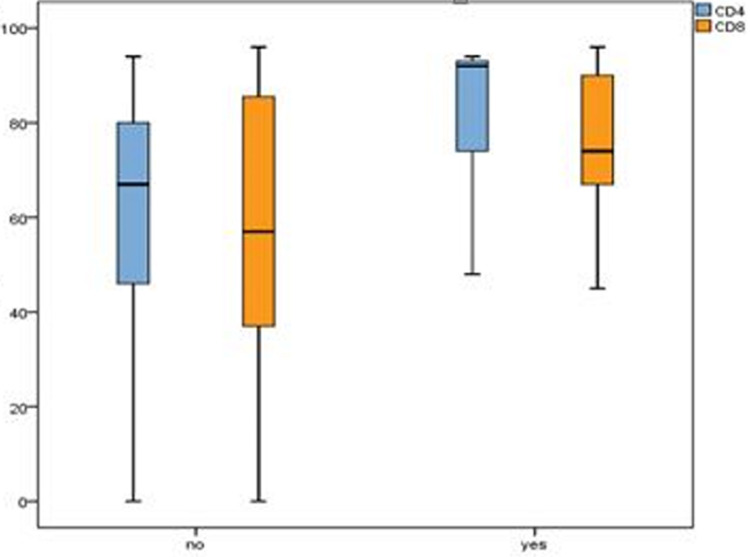
significantly increased mean levels of CD4+ and CD8+ by the presence of diabetes mellitus

In the complications leading to a fatal outcome, we encountered statistically significant differences between the group averages (p < 0.05). In general, complications were associated with relatively higher mean CD4+ and CD8+ levels, except for the group with sepsis and DAD pneumonia (Figure 4). On the other hand, the variant of the virus does not influence the average levels of CD4+ and CD8+ (p > 0.05). There is one exception to this trend and that is for cases with diabetes mellitus (Figure 5) presents the box plot of biomarkers considering the presence or absence of diabetes.

The presence or absence of comorbidities like atherosclerosis, arterial hypertension, liver cirrhosis and chronic ischemic heart disease could not be associated with significant changes in the observed mean levels of CD4+ and CD8+ (p > 0.05). However, the patients with diabetes mellitus have statistically significantly higher levels of biomarkers than those without this disease (p < 0.05) (Figure 5). For all groups by comorbidities, CD4+ means are higher than the CD8+.

The male group was associated with a significantly higher CD4+ biomarker mean compared to the female group (p < 0.05). Patients under the age of 60 have higher levels of biomarkers, but the difference compared to those over 60 is not statistically significant (Table 1).

## Discussion

During the acute phase of SARS-CoV-2, an adaptive immune response is required to control and eliminate the infection. For this reason, much research has been focused on cellular and humoral immunity in COVID-19, with the predominant studies reporting the presence of immune cells in the blood or in vitro stimulations [8]. The studies of CD4+ T helper cells, CD8+ cytotoxic T cells and immune cells in lung tissues in the individual phases of pneumonia and their interdependence with the developed complications are unique [9].

T cells are divided into two subpopulations: CD4+ T helper cells and CD8+ cytotoxic T cells. Both branches of cells contribute to the defense against respiratory viral infections. Activated CD8+ T cells control viral infections by eliminating virus-infected cells and secreting effector cytokines. In normal disease, this results in relief of symptoms and a milder disease course [10,11]. In this study, we found a dramatic decrease in both CD4+ and CD8+ in patients in the acute phase of pneumonia, between days 7 and 14, as well as in those who died of a septic complication. This fact allows us to conclude that the type of complication has a strong effect on the levels of both CD4+ and CD8+ cells. The sharp decline of CD4+ and CD8+ cells during the phase of fibrotic changes, as we observed, possibly contributes to the prolonged recovery of the lung. Another interesting inversely proportional statistical dependence was found: with increasing age, a decrease of 1.4 points in CD4+ and CD8+ was reported every year. This decline in immune cells may contribute to the more severe course of COVID-19 pneumonia as well as Long Covid.

The functional capacity of the cellular response plays a crucial role in determining clinical outcomes. High expression levels of effector molecules by CD8+ cytotoxic T cells during acute COVID-19 are associated with improved clinical outcomes [12]. Yang *et al*. 2020, found that CD4+ responses were slightly stronger than the CD8+ lymphocyte pool and may even increase over time, potentially reflecting antigenic resistance [13]. Schreibing *et al*. [14] also observed CD8+ T cell exhaustion in severe SARS-CoV-2 infection and identified a population of NK-like, terminally differentiated CD8+ effector T cells. Our results on lung tissue sections confirm the extreme downregulation of CD8+ during the acute period of the disease (including DAD and ARDS) with fatal outcomes.

A specific pool of CD4+ memory stem cells is thought to accumulate in COVID-19, which ensures durability of responses [15]. In our study, the value of CD4+ in desquamative pneumonia (DAD) was lower (49.42%) compared to ARDS (66.39%) and thrombosis cases (70.23%), suggesting that low levels of T cells in pneumonia contribute to fatal outcomes. Conversely, high expression of immune cells suggests a cytokine storm, ARDS, thrombosis and infarctions.

The presence or absence, as well as possible, complications of Atherosclerosis, Arterial Hypertension, Liver cirrhosis and Chronic Ischemic Heart Disease also could not be associated with significant changes in the observed mean levels of CD4+ and CD8+ (p > 0.05). No changes were found in the mean levels of CD4+ and CD8+ depending on the various co-morbidities, except for patients with diabetes mellitus, where significantly elevated mean levels are likely to cause cytokine storm, thrombosis, and infarctions.

Men and patients under 60 years of age, had higher mean levels of CD4+ and CD8+ cells. On the other hand, the lower mean levels of immune cells in women and patients over 60 years of age also explain the higher mortality in these groups. Females who died are twice as many, and those who died from COVID-19 over the age of 60 are nearly 6 times more than patients under the age of 60. Additionally, a statistically significant difference was observed in CD4+ values among patients below and above 60 years of age, in which CD4+ levels were higher in those below 60 years. Low levels of T cells in patients older than 60 years were associated with a worse prognosis. We found no statistical difference in the number of immune cells expressed by the two different variants of the Delta and Omicron viruses.

It has been known that older patients with lower lymphocyte counts were at a higher risk of severe disease and prolonged hospitalization [16]. Another study revealed a decrease in total lymphocytes, CD4+ T cells, CD8+ T cells, B cells, and NK cell counts. This study suggested that lymphopenia in CD8+ T cells could independently predict COVID-19 severity and treatment efficacy. It has been proposed that lymphocyte decrease is due to apoptosis or invasive migration from peripheral blood to the lungs, where high levels of viral replication occur [17]. However, our studies disprove these assumptions, showing that dramatic depletion of B and T lymphocytes in severe COVID-19 forms is also observed in lung tissue. Overall, the mean values of the three major lymphocyte subsets are reduced in patients with severe COVID-19, with T- and NK-cells below normal and B-cells completely exhausted. Within a few days after infection, a virus-specific immune response is ensured firstly by antibody-producing B cells. After recovery, B-cells become memory cells and provide long-term immunity. However, failure to activate B cells neutralizing antibodies can cause severe disease [18].

There is a significant correlation between the severity of COVID-19 and lymphopenia, which is associated with lymphocyte apoptosis induced by SARS-CoV-2 [19]. The presence of lymphopenia is usually considered a sign of a weak immune response to a viral infection. The correlation between lymphopenia and disease severity indicates that T and B cells play a key role in the pathology of COVID-19 [20]. Our study highlights that severe forms of SARS-CoV-2 infection are influenced not only by lymphopenia in the blood but also by the complete depletion of B cells in the tissues of affected patients. Our findings confirm the increased infiltration of CD68-infected macrophages in lung tissue, comprising 60-70% of the cellular composition, particularly prominent during the acute phase and initial stages of organization. However, in the fibrosis stage, depletion of macrophages is observed. Rubio-Casillas *et al*. [21] have proposed the hypothesis that IgG4 antibody blocks the binding of IgG3 to its Fc receptor and thus inhibits viral phagocytosis. The authors suggest that through this overexpression of IgG4, SARS-CoV-2 has developed a way to evade the immune system. We confirm this hypothesis in practice, since in severe COVID-19 with fatal outcomes and cases with ARDS, in the lungs of these patients there is an overexpression of IgG4 in 80-90%. In the organization and fibrosis phase, IgG4 levels drop to 5-10%.

## Conclusion

In this study, we aimed to investigate the levels of CD4+, CD8+, macrophages, NK, B lymphocytes and IgG4 expression in the lung tissue of deceased patients with fatal COVID-19, by determining their possible relationship in dynamics with the stages of pneumonia, correlation with concomitant diseases and immediate cause of death. The observed sharp decline in CD4+ and CD8+ in the acute phase of pneumonia and sepsis indicates exhaustion of the immune system. Conversely, high values in ARDS and thrombosis probably provoke cytokine storms and severe damage to the lungs, widespread thrombosis and infarctions. We did not report a statistically significant relationship of the levels of immune cells in relation to concomitant diseases, except in patients with Diabetes, where they were increased by 20%. With this study, we prove the hypothesis that extremely elevated IgG4 levels in the lung tissue are associated with the fatal outcome in severe COVID-19.

### What is known about this topic

- Till date, studies of CD4+, CD8+, macrophages, NK, B lymphocytes have been conducted on peripheral blood, in vitro models, or experimentally on mice;
- The plasma level of immune cells in the blood was measured in relation to comorbidity, age and severity of COVID-19;
- Rubio-Casillas, 2023, (21) has proposed the hypothesis that IgG4 antibody blocks the binding of IgG3 to its Fc receptor and thus inhibits viral phagocytosis.

### What this study adds

- This study reveals the infiltration of immune cells (B cells, CD4+, and CD8+, macrophages) in lung tissue in the four consecutive stages of COVID-19 pneumonia;
- The relationship of CD4+, CD8+ and immune cells were measured by comorbidities, complications, age, gender and virus variant;
- We prove the hypothesis that extremely elevated IgG4 levels in the lung tissue are associated with the fatal outcome of severe COVID-19.
